# Long-Lasting Effects of Incubation Temperature During Fetal Development on Subcutaneous Adipose Tissue of Broilers

**DOI:** 10.3389/fphys.2022.913496

**Published:** 2022-06-06

**Authors:** Ayla R. Almeida, Viviane S. Morita, João B. Matos Junior, Sarah Sgavioli, Tamiris I. Vicentini, Isabel C. Boleli

**Affiliations:** ^1^ Department of Animal Morphology and Physiology, Faculty of Agricultural and Veterinary Sciences, Sao Paulo State University—UNESP, Sao Paulo, Brazil; ^2^ Federal Institute of Acre—IFAC, Sena Madureira, Brazil; ^3^ Brasil University, Sao Paulo, Brazil

**Keywords:** adipose tissue cellularity, body composition, fat deposition, fetal programming, incubation temperature, lipid prolife

## Abstract

Increasing evidence indicates that fetal programming may cause permanent effects on offspring adipose tissue and body composition. Previous study showed reduction in newly-hatched broiler chick adiposity by manipulating incubation temperature during fetal development. The present study examined whether incubation temperature during fetal development has long-term effects on post-hatching fat deposition in broilers. Broiler breeder eggs (Cobb-500^®^) were incubated under constant low (36°C, LT), control (37.5°C, CT) or high (39°C, HT) temperature from day 13 onward, giving to eggshell temperature of 37.3 ± 0.08°C, 37.8 ± 0.2°C, and 38.8 ± 0.3°C, respectively. Male chicks were reared under recommended temperatures until 42 days old. LT 21 days old broilers exhibited higher blood cholesterol than CT broilers, and higher triglycerids, VLDL, and LDL, and lower HDL than CT and HT broilers. LT broilers presented higher liver cholesterol than CT broilers and lower ether extract percentage than CT broilers. Adipocyte count was lower in the abdomen than in the thigh. Until day 21 of age, feed intake was higher in LT than in HT broilers. At day 42 of age, blood cholesterol and LDL were higher in HT broilers than in CT and LT broilers. Liver cholesterol was higher in LT than in HT broilers. LT treatment reduced neck and increased thigh adipocyte size compared to CT treatment, while the HT treatment reduced abdomen and neck adipocyte size compared to other two treatments and in the thigh compared to LT treatment. In CT broilers, thigh adipocytes were smaller than abdomen and neck adipocytes. HT treatment increased adipocyte number per area in the neck compared to LT and CT treatment, and LT and HT treatments reduced adipocyte count in the thigh compared to CT treatment. CT broilers presented higher adipocyte count in the thigh than the abdomen and neck, while HT broilers presented higher adipocyte count in the neck than the abdomen and thigh. Cell proliferation was lower in the abdomen than in the thigh. The results show incubation temperature manipulation during fetal development has long-term and distinct effects on regional adiposity, and can be used to modulate broiler fat deposition.

## Introduction

Broiler lines genetically selected for higher and faster growth and higher feed efficiency and meat yield also exhibit greater liver growth and excessive adiposity (Tavárez and Solis de los Santos, 2016; Zaefarian et al., 2019) and higher sensitive to chronic heat stress (Abo Ghanima et al., 2020; Ibrahim et al., 2021). Due to its low thermal conductivity and increased insulator capacity, adipose tissue provides an insulating barrier to heat conduction and reduces the animal’s ability to respond to ambient temperature changes (Cooper and Trezek, 1971; Jequier et al., 1974; Savastano et al., 2009). Thus, although fat is important to structural and functional processes in the animal, excessive fat can be harmful depending on the environmental conditions to which the animal is exposed. It is well known that heat stress occurs when the ambient temperature exceeds the comfort zone for poultry species and/or metabolic heat production exceeds the bird’s ability to dissipate heat (Diarra and Tabuaciri, 2014; Akbarian et al., 2016). These studies indicate that very high body fat deposition can makes it difficult for broilers to lose body heat and maintain homeothermy when exposed to thermal stress, and this can lead to death. In addition, excessive fat is the main source of waste in the slaughterhouse and an important factor increasing costs (Tůmová and Teimouri, 2010). However, fat deposition and fatty acid composition in muscles contribute to attributes of meat quality, including juiciness, flavor, tenderness and other organoleptic properties (Wood, 1990; Wood et al., 1999; Jeremiah et al., 2003; Williams, 2007; Pereira and Vicente, 2013). Although the presence of fat in the muscle and skin confers high eating quality, and meat provides essential fatty acids and other nutrients for humans, there is an increased demand for high-quality meat consumption that is conducive to human and animal health and well-being, with the implication of a reduction in fat deposition in poultry (Wood et al., 2008; Del Gobbo et al., 2016; Forouhi et al., 2018). Thus, a reduction in fat deposition is desirable to the broiler meat production industry.

Increasing evidence suggests programming or imprinting of several tissues, including adipose tissue, takes place during fetal development, with long-term effects on post-born phenotype of mammals (Hanson and Gluckman, 2014). Tissue programming occurs during specific developmental windows, determined by developmental time throughout fetal development. In layer chickens and broilers, adipose tissue hyperplasia takes place during the second week of egg incubation (between E12 and E14), followed by a reduction of hyperplasia and increasing rates of hypertrophy through the subsequent days of incubation (Jo et al., 2009; Chen et al., 2014). In previous studies, we verified that manipulation of incubation temperature during fetal development affects skin thickness and vascularity, blood hormonal concentration and thermal preference of broiler chicks (Morita et al., 2016a; Morita et al., 2016b). We demonstrated that broiler chicks present differences in adipocyte size and proliferation across different regions of the body, and that continuously high incubation temperature during fetal development reduces abdominal and cervical adipocyte size (Almeida et al., 2016). These studies indicated fetal responsivity to manipulation of incubation temperature in broilers occurs from day 13. However, the application of this thermal manipulation during egg incubation to reduce or modulate post-hatch fat deposition in broilers has yet to be investigated.

The aim of the present study was to evaluate the long-term effect of manipulation of incubation temperature, from day 13, on adiposity of broilers after hatching. The effects of egg incubation at higher (39°C) and lower (36°C) incubation temperatures than the usual (37.5°C) on hyperplasia and hypertrophy of subcutaneous adipose tissue for three parts of the body, body weight and chemical composition, blood lipid profile and liver weight and lipid and cholesterol content of 21 and 42 day-old broilers was analyzed. External development of the avian embryo together with rapid prenatal and postnatal periods make broilers an excellent experimental model to investigate fetal tissue programming without maternal influences. Excessive adiposity compromises the health of many other animals, including domestic animals and humans, making long-lasting effects of incubation temperature during fetal development on subcutaneous adipose tissue of broilers studies on adipose tissue of interdisciplinary interest.

## Materials and Methods

### Experimental Procedures

A total of 900 fresh fertile eggs of 59-week-old broiler breeder flocks (Cobb500^®^) were obtained from a commercial hatchery (Globoaves, Itirapina, São Paulo, Brazil). All eggs were individually weighed before incubation. Eggs were then homogeneously distributed by weight (67–72 g) over six incubators (Premium Ecológica- IP200, Belo Horizonte, MG, Brazil) with automatic control of temperature and egg turning (1 turning per hour), kept at 37.5°C and 60% RH until day 12 of incubation. From day 13 to hatching day, incubation temperature protocols were similar to Almeida et al. (2016), i.e., the incubation temperature was maintained at 37.5°C (control), lowered to 36°C, or increased to 39°C, resulting in two incubators per incubation temperature profile. The average eggshell temperature under continuous incubation at 36°C (low temperature: LT), 37.5°C (control temperature: CT), and 39°C) high temperature: HT) from day 13 was 37.4 *±* 0.1°C, 37.8 *±* 0.1°C, and 38.8 *±* 0.3°C. Eggshell temperatures were measured due to its relationship with embryo temperature (van der Pol et al., 2014) and was measured every 30 min by thermistors (Alutal Type T; São Paulo, Brazil) attached to the equatorial region of the eggshells of 5 eggs per incubator. Data were recorded in data loggers connected to a computer. Manipulation of incubation temperature from the 13^rd^ day was based on the knowledge that functional maturation of the neuroendocrine hypothalamic-pituitary-thyroid axis is established around the 13^th^-14^th^ day of incubation (McNabb and Olsen, 1996; Jenkins and Porter, 2004), as well as the fetal timing of adipose tissue hyperplasia and hypertrophy (Jo et al., 2009; Chen et al., 2014). In all incubators, relative humidity was maintained at 60% throughout the incubation period, including hatching phase, to avoid effects on fetal development and survival. Incubation was considered finished after 504 h.

All chicks were removed from the hatchers and sexed by examining the feathers. Male chicks were then weighed and 115 chicks per incubation treatment were homogeneously distributed by average body weight (51 ± 2 g) into 5 replicate groups (23 chicks each). Only males were used because in previous studies we verified incubation temperature effects on thermal preference, skin thickness and vascularity, body surface temperature, and plasma concentration of T3 and GH of male broiler hatchlings (Morita et al., 2016b). The chicks were reared until 42 days post-hatching in thermal chambers containing 16 boxes each (2.5 × 1.5 m) and equipped with a monoblock refrigerant system, heaters, and exhaust fans, with automatic control of ambient temperature and a daily light regime (22L:2D) with the dark period occurring from 4 to 6 a.m. (Morita et al., 2020) and maintained at temperatures recommended for the strain (Cobb Broiler Management Guide, 2013). Average weekly values of temperature and RH in the climatic chambers were 33.3°C, 29.8°C, 27. 3°C, 25.4°C, 23.0°C, and 21.0°C and 65, 76, 68, 57, 65, and 51%, from the first to the 6 week of the experiment, respectively. Throughout the experimental period, broilers received water and feed *ad libitum*. The broilers received two diets based on corn and soybean meal formulated in accordance with the requirements established for broiler chickens by Rostagno et al. (2011): starter diet (1–21 days: ME = 2,883 Kcal/kg, PB = 21.27%) and grower diet (22–42 days: ME = 3,121 Kcal/kg, PB = 18.86%). The chicks were vaccinated against Marek and Poxvirus disease while in the hatchery, against infectious bursal disease (intermediate strain Lukert) and Newcastle (La Sota strain) on the 8th day post hatching and against infectious bursal disease (strong strain Australia v-877) on the 18th day.

### Blood Lipid Profile

Blood contents of cholesterol, triglycerides, high-density lipoprotein (HDL, low-density lipoprotein (LDL) and very low-density lipoprotein (VLDL) were analyzed at day 21 and 42. Blood samples were collected from 12 birds/incubation temperature/age by jugular puncture (two *ca* 3 ml samples per bird ) and stored in plastic tubes without anticoagulant for 1 h at room temperature. They were then centrifuged at 3,500 rpm and 4°C for 15 min for serum separation. After collection, serum (supernatant) was maintained in a freezer at −70°C until analysis. Measurements of total serum cholesterol, HDL, and triglycerides were performed using commercial kits (Labtest Diagnóstica S.A., Belo Horizonte, MG, Brazil). All serum samples were prepared and analyzed according to manufacturer’s specifications and three absorbance readings per birds/treatment with a spectrophotometer (Beckman Coulter, DU-800; Minnesota, United States) at a wavelength of 500 nm. VLDL and LDL concentrations were calculated by the equations: VLDL = Triglycerides/5; LDL = total cholesterol—(HDL + VLDL).

### Adipocyte Size, Number and Proliferation

On day 21 and 42 post-hatching, and after blood collection, male broilers were killed by cervical dislocation, and samples of adipose tissue collected from the abdomen, neck and thigh. The samples were fixed for 48 h in 10% formaldehyde at room temperature and then processed by standard histological procedures for optical microscopy. Briefly, samples were washed with distilled water, dehydrated in a series of increasing ethanol concentrations [70, 80, 90, 95, and 100% (2x)], diaphanized in a mixture of ethanol : xylene (1:1) and subsequently xylene (100%, 2x), infiltrated with a mixture of xylene:paraffin (1:1) followed by paraffin (100%), and embedding in paraffin (100%) (Almeida et al., 2016). For analysis of adipocyte size and number, deparaffinized semi-serial sections (7 μm thick) were rehydrated and stained with hematoxylin-eosin (HE) using standard procedures. Adipocytes area (μm^2^) and number (cell number/area) were determined using an image capture and analysis system (LAS Leica® type V4.3, Wetzlar, Hessen, Germany). Adipocyte area (μm^2^) was obtained by measuring 180 adipocytes per sample. Adipocyte number per area was counted on 10 microscopic fields with 202,800 μm^2^ each per sample. For adipocyte proliferation analysis, two 8-µm thick sections per sample were placed on electrically charged histological slides (Tissue-Tek R©AutoWrite R©Starfrost R©Green) and kept at 30°C until processing to allow them to settle on the slide. Histological sections were then submitted to an indirect immunohistochemical technique to mark and identify adipocytes expressing proliferating cell nuclear antigen (PCNA) protein (Starr Trek Universal HRP Detection System, Biocare Medical, Concord, California), following the manufacturer’s instructions (see Almeida et al., 2016). Briefly, after deparaffinization followed by hydration, histological sections were incubated with 15% hydrogen peroxide in methanol at room temperature for 30 min to inactivate endogenous peroxidase, washed with 1M Tris-HCl buffer pH 7.4 (3x for 1 min/each), and incubated for 90 min with non-specific horse serum (15 μL serum + 85 µL Tris-HCl buffer). After draining the non-specific serum, sections were incubated with primary monoclonal mouse anti-PCNA antibody (PCNA Clone PC10, 1:100 in Tris-HCl buffer, Concord, California) for 1 hour. After removing the primary antibody, sections were washed with Tris-HCl buffer, incubated with secondary antibody universal (Starr Trek Universal HRP Detection) at room temperature for 20 min and then washed with Tris-HCl buffer (2x, 2 min/each). Sections were then incubated with Avidin-HRP Trek at room temperature for 10 min and washed with Tris-HCl (2x for 2min/each). Following, diaminobenzidine-betazoid chromogen solution (DAB) [one drop (∼ 15 μl) + substrate buffer (4 μl), at room temperature for 3 min] was used to reveal antigen-antibody binding. The reaction was stopped by immersing the sections in distilled water and the sections were then dehydrated (ethanol 70, 80, 90, and 100%) and diaphanized (100% xylene, 2x), and the slides were mounted with Entellan. Negative controls were generated by omitting the section incubation with primary antibody. All procedures were performed in a humid chamber at 4°C. Mitotic rates were determined as the average number of adipocytes expressing PCNA present in 16 fields per sample (each field: 202,800 μm^2^) using the previously mentioned image capture and analysis system.

### Liver Lipids

Liver samples were obtained from the same birds used for blood and adipose tissue analyses (12 birds per treatment per age; 21 and 42 days old). Soon after collection, livers were weighed and divided in two, labelled and frozen and stored in a freezer at −20°C until analyses. Total lipid content was determined following the protocol established by Bligh and Dyer (1959). Cholesterol content was determined by enzymatic measurement (Saldanha et al., 2004). Briefly, 4 ml of KOH 50% and 6 ml of ethanol were added to 2 g of liver in falcon tube. Tubes containing the samples were kept in water bath at 40°C for 2 h, followed by an additional 10 min at 60°C. 5 ml distilled water was then added to each tube. For cholesterol extraction, 10 ml of hexane was added and vortexed for 1 min. After allowing separation of the mixtures into two phases, the hexane phase was removed and transferred to another tube. This extraction procedure was repeated three times. 3 ml of the hexane extract was then dried in N_2_ and 0.5 ml of isopropanol was added to dissolve the residue. Following the procedures recommended by the enzymatic method cholesterol kit (Cholesterol cod.74305 Laborclin, Pinhais, PR, Brazil), absorbance readings were made at 499 nm in a spectrophotometer (Beckman Coulter DU-800- GMI, Minnesota, United States).

### Body Weight and Chemical Composition

At day 21 and 42 after hatching, an additional 12 broilers/incubation temperature were weighed and killed by stunning with CO_2_ followed by bleeding, and stored at −20°C before analyses of moisture, dry matter, ether extract, crude protein, and ash. Broilers were ground and a homogeneous sample dried at 55°C for 4 days, and remaining pre-dried matter weighed, ground and homogenized. After pre-drying, samples (1 g each) were dried at 105°C for 16 h and the remaining dry matter weighed. The moisture was calculated as the total difference between wet and dry weights. Moisture and dry matter were given in total grams and percentage in relation to body weight. For ash content determination, the dried samples were heated in a muffle furnace at 600°C for 4 h. Organic matter was calculated as the difference between dry matter and ash weights. Ether extract content (crude fat) was determined by extraction with petroleum ether for 8 h using 1 g of dry matter per sample and corresponded to difference between the weights before and after extraction. Following the crude fat extraction, the fat-free dry matter (100 mg per samples) was used for crude protein determination by the Kjeldahl method for detection of total N content, and crude protein was calculated as N content x 6.25. Two replicates per bird of broiler body composition were used following the methods of Silva and Queiroz (2002). Crude protein, ether extract and ash contents are expressed in g and % in relation to dry matter contents.

### Broiler Performance

The broiler body weights were determined at 21 and 42 days of age (g), and feed intake (g), weight gain (g) and feed conversion (g/g) for two periods: 1–21 and 22–42 days were calculated. Feed intake was calculated by difference between the amounts of feed offered and uneaten feed in each replicate. Weight gain corresponded to the difference between body weight at the beginning and end of the rearing period. Feed conversion was calculated by dividing the feed consumed by the body weight gain (gram per gram).

### Statistical Analyses

Serum lipid profile, body weight and chemical composition, total lipid and cholesterol concentrations in the liver, and performance, were analyzed, comparing the effects of the three incubation temperatures (IT: 36°C, 37.5°C and 39°C) on the grow out phase. The model Y = μ + IT + *e*, in which Y is the dependent variable, μ is the overall mean and *e* is the residual error, was applied to the data. Adipocyte size and proliferation rate were analyzed with respect to the effects of incubation temperatures (IT) and body regions (BR) (neck, abdomen and thigh) and of the respective interactions (IT x BR), using the following model: Y_ijk_ = µ + IT_i_ + BR_j_ + (IT x BR)_ij_ + *e*_ijk,_ where µ is the overall mean and *e*_ijk_ is the residual error term. All data were analyzed using the GLM procedure of SAS (SAS-Institute Inc, 2009). When necessary, differences among means were tested by Tukey`s test. In all cases, *p*-values ≤ 0.05 were considered statistically significant.

## Results

### Serum Lipid Profile and Liver Lipid and Cholesterol Levels

At day 21 of age, broilers from eggs incubated at 36°C exhibited higher serum triglycerides, VLDL and LDL concentrations and lower HDL concentration than broilers reared from eggs incubated at 37.5°C or 39°C, that did not differ, as well as higher cholesterol concentration than broilers from egg incubation at 37.5°C (Table 1). At 42 days of age, incubation temperature influenced cholesterol and LDL concentrations only, values were higher in broilers from egg incubated at 39°C than at 37.5 and 36°C, which did not differ. Incubation temperature had no significant effect on the weight and lipid concentration of the liver at 21 or 42 days of age. However, incubation temperature changed the liver cholesterol concentration, which was higher in 21 day-old broilers reared from eggs incubated at 36°C than at 37.5°C and in 42 day-old broilers reared from eggs incubated at 36°C than 39°C (Table 2).

**TABLE 1:** Blood lipid profile of male broilers at day 21 and 42 of age, according to incubation temperatures.

Variables	Incubation temperatures			*p*-values	CV(%)
	36°C	37.5°C	39°C		
21 d. old broilers					
Cholesterol	184.79 ± 29.3^A^	141.07 ± 28.6^B^	159.62 ± 18.0^AB^	0.0372	16.04
Triglycerids	39.54 ± 3.2^A^	30.63 ± 3.8^B^	31.03 ± 5.9^B^	0.0015	21.42
VLDL	7.91 ± 1.7^A^	6.12 ± 0.8^B^	6.21 ± 3.2^B^	0.0001	21.43
HDL	62.61 ± 24.4^B^	99.47 ± 18.4^A^	92.31 ± 16.5^A^	0.0088	23.45
LDL	114.27 ± 39.6^A^	35.48 ± 23.6^B^	61.11 ± 24.8^B^	0.0008	45.02
42 d. old broilers					
Cholesterol	126.51 ± 30.93^B^	115.34 ± 22.51^B^	153.97 ± 20.6^A^	0.0013	15.44
Triglycerids	35.54 ± 5.54	35.95 ± 6.68	34.09 ± 9.3	0.8184	21.77
VLDL	7.11 ± 3.2	7.18 ± 1.15	6.82 ± 2.0	0.8147	21.78
HDL	71.43 ± 18.7	60.84 ± 17.98	71.56 ± 19.2	0.2090	20,78
LDL	47.98 ± 5.3^B^	47.32 ± 6.2^B^	75.59 ± 1.,2^A^	0.0073	32.56

^A−B^: Means with distinct superscript letters differ significantly (rows) (*p* < 0.05).

**TABLE 2:** Liver weight and lipid and cholesterol contents of male broilers at 21 and 42 days of age, according to incubation temperatures.

Variables	Incubation temperatures			*p*-values	CV(%)
	36°C	37.5°C	39°C		
21 d. old broilers					
Liver Weight (g)	20.9 ± 3.4	20.7 ± 2.9	20.6 ± 3.6	0.4055	16.87
Liver Lipids (%)	4.8 ± 1.2	4.5 ± 1.3	4.3 ± 1.7	0.7851	29.92
Liver Cholesterol (µg/ml)	108.8 ± 18.9^A^	75.6 ± 2.7^B^	89.5 ± 16.8^AB^	0.0028	16.61
42 d. old broilers					
Liver (g)	40.8 ± 5.2	36.03 ± 4.8	34.7 ± 5.1	0.2343	16.87
Liver Lipids (%)	4.1 ± 1.3	3.35 ± 1.5	3.70 ± 0.7	0.5519	30.52
Liver Cholesterol (µg/ml)	136.9 ± 13.6^A^	123.2 ± 10.9^AB^	106.8 ± 12.2^B^	0.0025	16.05

^A−B^: Means with distinct superscript uppercase letters differ significantly (rows) (X ± SD, *p* < 0.05).

### Adipocyte Size, Number and Proliferation Rate

At day 21 of age, no significant effects of incubation temperature, body region and their interaction were observed on adipocyte size, number per area. Incubation temperature did not affect mitotic rate, and, independently of treatment, the thigh presented higher cell proliferation than the abdomen (*p* < 0.05; Table 3). At 42 days of age, significant effects of body region for cell proliferation remained apparent, with significantly higher mitotic rate in the thigh than in the abdomen. Significant interactions between incubation temperature and body region for adipocyte area and number per area (Table 3) also occurred. As showed in Table 4, adipocyte area in the abdomen was smaller in broilers from eggs incubated at 39°C than at 36°C or 37.5°C, which did not differ. Adipocyte area in the neck was also smaller in broilers from eggs incubated at 39°C than at 36°C or 37.5°C, and also smaller in broilers from eggs incubated at 36°C than at 37.5°C. In the thigh, adipocyte area was larger in broilers from egg incubated at 36°C than at 37.5°C or 39°C, which did not differ. Significant regional differences in adipocyte size only occurred in broilers from eggs incubated at 37.5°C. In these broilers, the area of thigh adipocytes was smaller than the area of abdomen and neck adipocytes, which did not differ. Incubation temperature influenced adipocyte number per area in the neck and thigh, with higher adipocyte number in the neck in broilers from egg incubated at 39°C than at 37.5°C or 36°C, which did not differ. In the thigh, adipocyte number was similar in broilers from eggs incubated at 36°C and 39°C, and both presented lower adipocyte number compared to broilers of eggs incubated at 37.5°C. In addition, no significant difference in adipocyte number was observed among the body regions in broilers from egg incubated at 36°C. However, in broilers from eggs incubated at 37.5°C, adipocyte was more numerous in the thigh than in the other two body regions, that did not differ. In broilers from egg incubated at 39°C, adipocyte number was larger in the neck compared to in the abdomen and thigh, which did not differ.

**TABLE 3:** Adipocyte size and counts (number per area) and proliferation rate of 21 and 42 d old male broilers, according to incubation temperature and body region.

	Area (µm^2^)	Count (cells/area)^1^	Proliferation (cells/area)^2^	Area (µm^2^)	Count (cells/area)^1^	Proliferation (cells/area)^2^
**21 d-old broilers**					**42 d-old broilers**	
Incubation Temperature (IT)						
36°C	1,419 ± 463	82 ± 35	18 ± 6	6,209 ± 1,602	75 ± 12	9 ± 3
37.5°C	1,332 ± 169	92 ± 21	18 ± 6	5,112 ± 3,320	96 ± 35	10 ± 3
39°C	1,472 ± 426	102 ± 30	18 ± 6	1,950 ± 1,010	83 ± 27	9 ± 3
Body Region (BR)						
Abdomen	1,496 ± 466	78 ± 25^b^	18 ± 4	5,584 ± 2,424	78 ± 11	8 ± 2^b^
Neck	1,464 ± 283	87 ± 22^ab^	17 ± 7	4,875 ± 3,013	81 ± 24	9 ± 2^ab^
Thigh	1,273 ± 336	106 ± 36^a^	19 ± 6	2,763 ± 2,536	95 ± 37	11 ± 3^a^
*p*-values						
IT	0.4082	0.3938	0.9420	<0.0001	0.0140	0.5724
BR	0.1363	0.0594	0.7695	<0.0001	0.0152	0.0064
IT x BR	0.1612	0.2082	0.8253	<0.0001	<0.0001	0.9775
CV (%)	26	31	27	23	20	27

1 Average adipocyte number counted in 10 areas of 202,800 μm^2^.

2 Average number of adipocytes presenting immunostained nuclei for PCNA (proliferating cell nuclear antigen) counted in 16 areas of 202,800 μm^2^.

^a-b^: Means with distinct superscript lowercase letters differ significantly (columns) (X ± SD, *p* < 0.05).

**TABLE 4:** Unfolding of the interaction between incubation temperatures and body regions on the adipocyte area and number of 42 d. old male broilers.

Body regions	Incubation temperatures			*p*-values
	36°C	37.5°C	39°C	
Adipocyte Area (μm^2^)				
Abdomen	6574.44^Aa^	7310.80^Aa^	2436.12^Ba^	<0.0001
Neck	5460.24^Ba^	7841.20^Aa^	1322.70^Ca^	<0.0001
Thigh	6970.92^Aa^	1001.99^Bb^	2247.72^Ba^	<0.0001
*p*-values	0.0557	<0.0001	0.1950	
Adipocyte Number (nº cells/area)^1^				
Abdomen	85.50^Aa^	74.67^Ab^	74.61^Ab^	0.3356
Neck	65.99^Ba^	74.43^Bb^	106.80^Aa^	0.0004
Thigh	78.34^Ba^	139.27^Aa^	71.67^Bb^	<0.0001
*p*-values	0.2230	<0.0001	0.0018	

1 Average adipocyte number counted in 10 areas of 202,800 μm^2^.

^A−B, a-b^: Means with distinct superscript uppercase (rows) and lowercase (columns) letters differ significantly (*p* < 0.05).

### Body Chemical Composition

Ether extract content was the only variable influenced by incubation temperature in 21 days old broilers (Table 5). Ether extract content was lower in broilers from eggs incubated at 36°C than at 37.5°C and 39°C, and these did not differ. 42 days after hatching no significant effect of incubation temperature on the analyzed variables was found.

**TABLE 5:** Body weight and composition of male broilers at day 21 and 42 of age, according to incubation temperature.

Variables	Incubation temperatures				*p*-values	CV(%)
		36°C	37.5°C	39°C		
21 d. old						
Body Weight	(g)	822 ± 33	799 ± 24	802 ± 22	0.3968	24.47
Dry matter	(g)	256.44 ± 13.5	259.97 ± 19.6	255.29 ± 16.5	0.3037	12.58
	(%)^1^	31.20 ± 0.6	32.52 ± 0.4	31.83 ± 0.7	0.2110	1.17
Moisture	(g)	565.24 ± 21.4	539.01 ± 12.6	546.93 ± 19.2	0.2037	21.42
	(%)^1^	68.80 ± 0.6	67.47 ± 0.4	68.17 ± 0.7	0.2110	1.17
Ash	(g)	19.98 ± 1.1	17.12 ± 2.3	16.46 ± 3.1	0.1027	2.77
	(%)^2^	7.79 ± 0.2	6.58 ± 0.7	6.47 ± 1.3	0.5098	1.07
Ether Extract	(g)	69.28 ± 5.8	81.00 ± 12.1	72.63 ± 6.4	0.2577	9.75
	(%)^2^	27.02 ± 1.7^B^	31.04 ± 2.3^A^	28.42 ± 2.1^AB^	0.0448	2.71
Crude Protein	(g)	150.83 ± 6.6	149.97 ± 7.5	148.09 ± 7.7	0.8343	6.86
	(%)^2^	58.84 ± 1.1	57.80 ± 2.5	58.09 ± 4.3	0.3210	3.02
42 d. old						
Body Weigth	(g)	2.385 ± 124.0	2.355 ± 97.6	2.305 ± 109.1	0.4168	5.43
Dry Matter	(g)	975.65 ± 54.4	937.43 ± 100.7	914.83 ± 55.2	0.4089	9.83
	(%)^1^	40.92 ± 1.2	39.29 ± 2.7	39.68 ± 4	0.4942	2.16
Moisture	(g)	1409.12 ± 81.9	1447.18 ± 17.8	1390.26 ± 103.2	0.5225	76.53
	(%)^1^	59.08 ± 1.2	60.71 ± 2.7	60.32 ± 2.4	0.4942	2.16
Ash	(g)	60.91 ± 6.3	55.56 ± 5.2	55.20 ± 4.7	0.2245	5.76
	(%)^2^	6.24 ± 0.3	5.99 ± 1.0	6.04 ± 0.4	0.8279	0.61
Ether Extract	(g)	329.69 ± 36.9	294.82 ± 89.2	264.14 ± 54.1	0.3206	6.70
	(%)^2^	33.86 ± 4.1	30.98 ± 6.4	28.66 ± 4.3	0.3399	5.41
Crude Protein	(g)	1305.20 ± 144.8	1358.01 ± 96.6	1310.63 ± 244.8	0.3485	16.55
	(%)^2^	54.84 ± 2.57	58.83 ± 5.96	54.87 ± 4.85	0.6410	7.25

^1,2^: Calculated with respect to body weight and dry matter, respectively.

^A−B^: Means with distinct superscript uppercase letters differ significantly (rows) (*p* < 0.05).

### Broiler Performance

Table 6 presents feed intake, weight gain, body weight and feed conversion obtained for two rearing periods: 1–21 days and 21–42 days of age. Feed intake until 21 days of age was the only variable significantly affected by incubation temperature, and this was lower for broilers from eggs incubated at 39°C than 36°C.

**TABLE 6:** Broiler body weight at 21 and 42 days of age and performance from day 1 to 21 and from day 22 to 42 of age, according to incubation temperature.

Variables	Incubation temperatures			*p*-values	CV(%)
	36°C	37.5°C	39°C		
1–21 days of age					
Feed Intake (g)	983 ± 54^A^	953 ± 100^AB^	902 ± 55^B^	0.0317	52.51
Weight Gain (g)	757 ± 48	738 ± 41	758 ± 52	0.5099	26.41
Body Weight (g)	822 ± 48	799 ± 104	802 ± 52	0.3967	27.47
Feed Conversion (g/g)	1.30 ± 0.12	1.29 ± 0.11	1.22 ± 0.21	0.7493	0.75
22–42 days of age					
Feed Intake (g)	2,729 ± 261	2,798 ± 300	2,818 ± 434	0.9123	10.02
Weight Gain (g)	1,515 ± 115	1,538 ± 91	1,455 ± 82	0.4081	7.07
Body Weight (g)	2,385 ± 123	2,325 ± 107	2,245 ± 82	0.4168	5.43
Feed Conversion (g/g)	1.81 ± 0.18	1.82 ± 0.11	1.94 ± 0.31	0.5734	11.57
1–42 days of age					
Feed Intake (g)	3,713 ± 286	3,751 ± 333	3,720 ± 422	0,6217	6,97
Weight Gain (g)	2,272 ± 124	2,275 ± 107	2,196 ± 81	0,5365	5,21
Feed Conversion (g/g)	1.63 ± 0.12	1,65 ± 0.08	1,69 ± 0.19	0,5945	8,98

^A−B^: Means with distinct superscript uppercase letters differ significantly (rows) (*p* < 0.05).

## Discussion

Low-fat deposition in broilers is currently desirable to preserve and/or to promote their well-being and survival in the face of heat stress, to increase feed-to-muscle conversion, to maximize production efficiency of the industry, and to address the market’s requirement for lean meat. Previous study showed continuously high incubation temperature during fetal development reduces abdominal and cervical subcutaneous adipocyte hypertrophy in broiler hatchlings (Almeida et al., 2016), causes variations in subcutaneous adipose tissue deposition across different body areas and in skin temperature and thickness of broiler hatchlings (Bai et al., 2015; Almeida et al., 2016; Morita et al., 2016b). The current study assessed whether low (36°C) and high incubation temperatures (39°C) from day 13, exerted a long-term and regional-related effect on fat deposition in broilers by analyzing subcutaneous adipose tissue hyperplasia and hypertrophy in three anatomically distinct adipose deposits (neck, abdomen, and thigh), liver weight and content of lipid and cholesterol, blood lipid profile, body weight and chemical composition, and performance of 21-day-old and 42-day-old male broilers.

We evaluated the effects of incubation temperature on regional cellularity pattern of adipose tissue depots by measuring adipocyte size and number per area. Effects of incubation temperatures and differences among the three body regions on these adipocyte characteristics were apparent in 42-day-old broilers. At this age, egg incubation at 39°C reduced the abdominal adipocyte size by approximately 67% compared to egg incubation at 36°C or 37.5°C, but without change in the number of adipocytes per area. It also reduced neck adipocyte size by approximately 83 and 76% compared to 37.5°C and 36°C, respectively, resulting in an increase in the adipocyte number per area by approximately 43 and 38% under incubation temperature of 37.5°C and 36°C, respectively. These findings showed that high incubation temperature greatly reduced the hypertrophic growth of adipocytes in the abdomen and neck, with much more intense effects on fat deposition and cell density (cells per area) in the adipose tissue of the neck. In contrast to that observed for the abdomen and neck, no significant effect of high incubation temperature on adipocyte size was recorded in the thigh. However, there was an increase in the cell density of the thigh adipose tissue, as showed by the increase in the number of adipocytes/area. These region-specific effects of high incubation temperature may be related to distinct metabolic rate existing among the body adipose tissue depots (Xiao et al., 2019). Contrary to the high incubation temperature, low incubation temperature (36°C) did not influence the abdominal adipocyte size nor the counts, and markedly increased thigh adipocyte size, which was about 86 and 68% greater compared to 37.5°C or 39°C, respectively, resulting in a decrease of approximately 56% in the cell density compared to 37.5°C. However, like 39ºC, egg incubation at 36°C also reduced the size of neck adipocytes, but its effect was less pronounced because adipocytes were only approximately 30% smaller compared to 37.5°C. Since adipocyte hypertrophy is directly related to fat deposition (Tchernof et al., 2006; Kou et al., 2012; Choe et al., 2016), this supports the idea that high incubation temperature induced a decrease in fat deposition in neck and abdominal adipocytes, while a low incubation temperature induced increased deposition in thigh and abdominal adipocytes and a decreased deposition in neck adipocytes. From a developmental view, our results are of great interest and importance because they reveal a close link between egg incubation temperature during fetal development and postnatal adiposity in broilers. This leads us to recognize adipose tissue as a key target of fetal developmental programming for birds, as has been observed for mammals (Ravelli et al., 1999; Lecoutre et al., 2019; Lecoutre et al., 2021). We previously reported abdominal and/or cervical adipocyte size reduction in response to egg incubation at 36°C or 39°C in broiler hatchlings (Almeida et al., 2016). Thus, as expected, the present study confirmed that incubation temperature during fetal development has a long-term effect on adiposity of broilers. The occurrence of effects of incubation temperature on adipose tissue only at 42 days of age may be related to a significantly higher cumulative fat deposition between 21–42 days than between 1–21 days of age (Hen et al., 2014). Comparison among the three body regions showed broilers reared from eggs incubated at 37.5°C had similar size and number of adipocytes per area in the abdomen and neck, but these two regions presented adipocyte size approximately 86–87% larger and adipocyte number per area 50 % smaller than the thigh, indicating higher adipocyte hypertrophy in the abdomen and neck adipose tissue than the thigh. When eggs were incubated at 36°C or 39°C, no differences in the adipocyte size and number per area were found among the three body adipose depositional sites, showing the same hypertrophy rate for the three regions.

In this study, we evaluated the effects of incubation temperature on regional subcutaneous adipocyte hyperplasia by analyzing adipocyte proliferation. At 42 days of age, independently of incubation temperature, differences among the three regions were apparent. The rate of adipocyte proliferation was lower in the abdomen than the thorax and thigh (abdomen > thigh). An effect of incubation temperature on adipocyte proliferation was also not previously found in broiler hatchlings, but in contrast to what we observed for 42 day-old broilers, the cervical region exhibited higher cell proliferation than the abdomen and thigh (Almeida et al., 2016). Regional differences in adipocyte size and proliferation was reported for chicken and/or broiler lines in other studies (Guo et al., 2011; Chen et al., 2014; Xiao et al., 2019). Heterogeneity in adipocyte size among adipose tissue deposits also occurs in mammals, including humans (Sbarbati et al., 2010). However, to the best of our knowledge, for the first time in the literature, comparison between body regions revealed that manipulation of incubation temperature during fetal development also reprograms differences in size and number of adipocytes per area among different adipose tissue deposits in later life of broilers.

Temporal analysis showed, from day 21 to 42 of age, broilers reared from eggs incubated at 36°C, 37.5°C, and 39°C increased their adipocyte area by approximately 437, 384, and 132% and reduced rate of adipocyte proliferation by approximately 50, 45, and 50%, respectively. During the same period, differences in the rate of growth and proliferation of adipocytes were also recorded for regional subcutaneous adipose tissue deposits: the size of adipocytes increased by 273% in the abdomen, 233% in the neck and 117% in the thigh, while the proliferation of adipocytes decreased around 55% in the abdomen, 47% in the neck and 42% in the thigh. This showed that, independently of incubation temperature and body region, both hyperplasia and hypertrophy contributed to adipose tissue growth throughout the broiler age, in agreement with Cartwright (1991), but the magnitude of contribution of hyperplasia was larger than hypertrophy from day 21. Our data differ from those obtained for chicken by Cartwright (1991), who demonstrated adipocyte hyperplasia and hypertrophy increase with age. They differ from those reported for goose by Huo et al. (2021), who verified the average number of adipocytes had no increase after birth. We previously reported the adipocyte size and proliferation rate in abdominal, cervical and thigh adipose deposits of broiler hatchlings from eggs incubated at 36°C, 37.5°C, and 39°C in Almeida et al. (2016). Comparing the data obtained in the present study with those in Almeida et al. (2016), we verified that broilers have a low rate of adipocyte hypertrophy and a marked increase of adipocyte hyperplasia from hatching to 21 days of age, in contrast to what occurs between 21 and 42 days of age. White adipose tissue is characterized by its capacity to adapt and expand, or not, in response to energy availability or demand by changing adipocyte hypertrophy and hyperplasia (Öst and Pospisilik, 2015; Pellegrinelli et al., 2016). Positive links between adipose tissue growth by hyperplasia and hypertrophy and body weight was recorded in chickens (*Gallus domesticus*) (Cartwright, 1991). Despite that, in the present study, no incubation temperature-related changes in broiler body composition (ether extract, crude protein, ash, water contents) at 21 and 42 days of age and in performance (body weight, feed intake, feed conversion, body gain) that showed a relationship with marked hyperplasia and reduced hypertrophy after 21 days of age, were found. However, although cumulative fat deposition in broilers is greater at 21–42 days than at 1–21 days of age, the weekly rate of fat deposition decreases with age after 21 days (Henn et al., 2014). Furthermore, broilers decrease their activity levels with age (Yang et al., 2020), which has been associated with increased resting and high locomotion-associated metabolic costs, and results in heavier and less active broilers (Tickle et al., 2018). Thus, independently of incubation temperature during fetal development, the occurrence of increased adipocyte hyperplasia and reduced adipocyte hypertrophy in the three adipose deposits from day 21–42 of age that were observed in the present study appear to result from simultaneous increased energy availability and decreased metabolic expenses associated with locomotion, as a function of age.

Lipid droplets of an adipocyte are composed of a hydrophobic core of triacylglycerides and cholesterol esters, which, in birds, result from *de novo* lipogenesis occurring predominantly in the liver, where glucose is catabolized to acetyl-CoA. This is subsequently converted into fatty acids and cholesterol, incorporated into very low density lipoproteins (VLDLs) and transported to other tissues, including adipose tissue, via blood circulation (Walther and Farese, 2012; Wang et al., 2017). We further analyzed blood lipid profile and liver weight and cholesterol and lipid contents to assess the effects of incubation temperature on lipid metabolism. Egg incubation at 36°C and 39°C influenced blood and liver lipid profile of 21 and 42 day-old broilers. At 21 days of age, a low egg incubation temperature (36°C) increased liver cholesterol content compared to 37.5°C, as well as increased the blood contents of cholesterol, triglycerids, VLDL, and LDL and diminished blood HDL content compared to 37.5°C and 39°C. These effects may be a result of the increase in energy intake due to higher feed consumption from day 1 to 21 exhibited by broilers reared from eggs incubated at 36°C. Although these data indicate an increase in broiler lipid metabolism induced by low incubation temperature (36°C), this increase was not converted into larger adipocyte size and proliferation at day 21 in any of the three subcutaneous adipose tissue deposits, as appears to be demonstrated by the absence of incubation temperature-dependent differences in the area and proliferation of adipocytes at this age. However, Almeida et al. (2016) demonstrated that broiler hatchlings from eggs incubated at 36°C exhibited smaller adipocytes in the neck than the abdomen and leg, due to the higher rate of cell proliferation reported in the neck. This led us to raise the possibility that the absence of differences in adipocyte size and proliferation between regional adipose tissue deposits and incubation temperatures observed in 21-day-old broilers results from an increase in lipid metabolism, from hatchling to 21 days age, as indicated by the highest values recorded for lipid profile at this age. 42 days after hatching, liver cholesterol content decreased as incubation temperature increased, resulting in lower cholesterol values in broilers from eggs incubated at 39°C than at 36°C. In contrast, broilers hatched from eggs incubated at 39°C exhibited increased blood cholesterol and LDL contents compared to broilers hatched from eggs incubated at 36°C or 37.5°C. At this age, adipocyte size in the three body regions analyzed were significantly smaller in broilers reared from eggs incubated at 39°C than at 36°C and 37.5°C, and there was no difference in the rate of cell proliferation between broilers hatched from eggs incubated at different temperatures (our data). Thus, our results suggest a lack of links between increased blood cholesterol and LDL and liver cholesterol with adipocyte size at 42 days of age when egg incubation occurred at 39°C. Lipids are important structural and functional components of animal cells, particularly playing an important role in membrane stabilization, permeability and signaling (Santos and Preta, 2018). As a component of cell membranes, cholesterol is a neutral lipid that acts as a buffering molecule of membrane fluidity despite deviations in body temperature. Membrane stability is required for the continued action of membrane proteins in the transport of molecules across the lipid bilayer. Cholesterol decreases membrane fluidity at high temperatures and increases fluidity at low temperatures (Kroes and Ostwald, 1971; Bloom and Mouritsen, 1988; Yeagle et al., 1988). This makes cholesterol an essential molecule for cell proliferation during growth and cell renewal. Consequently, temperature an important determinant of animal cholesterol content and high ambient and acclimation temperatures induce an increase in cholesterol content, while low temperatures induces a reduction (e.g., Crockett, 1998; Hassett and Crockett, 2009). A link between ambient temperature and blood lipid profile has been also reported. Halonen et al. (2011), for example, found decreased HDL levels and increased LDL level in association with each 5°C increase in ambient temperature. To our knowledge, the long-term associations between egg incubation temperature during fetal development and blood lipid profile and total liver cholesterol have not been previously reported for broilers or other birds.

In conclusion, our data demonstrate that incubation temperature during the fetal phase 1) reprograms postnatal ontogenetic development of adipose tissue and lipid metabolism and 2) influences the hypertrophy and hyperplasia rate of regional adipocyte deposits in different ways. These findings on the occurrence of fetal reprogramming of postnatal adipose tissue development point to the potential use of manipulation of incubation temperature as a management for modulation of adiposity in broiler lines.

## Data Availability

The raw data supporting the conclusions of this article will be made available by the authors, without undue reservation.
